# Self-organizing maps as an approach to exploring spatiotemporal diffusion patterns

**DOI:** 10.1186/1476-072X-12-60

**Published:** 2013-12-23

**Authors:** Ellen-Wien Augustijn, Raul Zurita-Milla

**Affiliations:** 1 Faculty of Geo-Information Science and Earth Observation, University of Twente, Enschede, The Netherlands

## Abstract

**Background:**

Self-organizing maps (SOMs) have now been applied for a number of years to identify patterns in large datasets; yet, their application in the spatiotemporal domain has been lagging. Here, we demonstrate how spatialtemporal disease diffusion patterns can be analysed using SOMs and Sammon’s projection.

**Methods:**

SOMs were applied to identify synchrony between spatial locations, to group epidemic waves based on similarity of diffusion pattern and to construct sequence of maps of synoptic states. The Sammon’s projection was used to created diffusion trajectories from the SOM output. These methods were demonstrated with a dataset that reports Measles outbreaks that took place in Iceland in the period 1946–1970. The dataset reports the number of Measles cases per month in 50 medical districts.

**Results:**

Both stable and incidental synchronisation between medical districts were identified as well as two distinct groups of epidemic waves, a uniformly structured fast developing group and a multiform slow developing group. Diffusion trajectories for the fast developing group indicate a typical diffusion pattern from Reykjavik to the northern and eastern parts of the island. For the other group, diffusion trajectories are heterogeneous, deviating from the Reykjavik pattern.

**Conclusions:**

This study demonstrates the applicability of SOMs (combined with Sammon’s Projection and GIS) in spatiotemporal diffusion analyses. It shows how to visualise diffusion patterns to identify (dis)similarity between individual waves and between individual waves and an overall time-series performing integrated analysis of synchrony and diffusion trajectories.

## Background

Spatiotemporal analysis of epidemic waves can reveal important information on anomalies and trends, and provide inside into the underlying diffusion patterns [1,2]. These patterns are categorised as contagious spread, hierarchical spread, or mixed diffusion. Contagious spread depends on direct person to person contact and results in centrifugal patterns from the source outward [2]. Hierarchical spread refers to disease transmission through an ordered sequence of geographic locations (normally based on their size) [1] and it can be related to the movement of people, carrying a disease to a new centre of population via long distance travel. Due to this, hierarchical spread is typically characterized by the display of synchrony among locations that have similar size but that are geographically apart [2]. Two or more locations are synchronized when they exhibit a parallel development in the number of disease cases.

The search for synchrony is not unique to epidemiology but originates in innovation diffusion and ecology, and it occurs in many other disciplines [3]. Hence, multiple methods exist to quantify and to map synchrony [4]. Among these methods wavelets are frequently used as they also allow to study non-stationary (trends) in time series [5]. Wavelets analyse disease diffusion in the frequency domain where synchrony can be identified via the coherence in the phase of the number of diseases cases at each geographic location [6].

Besides synchrony, another important property of spatiotemporal disease diffusion is the trajectory of wave propagation. This trajectory captures the step by step diffusion by describing the speed and direction of spread [2]. As waves of infectious diseases are normally a combination of contagious and hierarchical spread [2], this trajectory is not a single and continuous line (as a trajectory representing human movement) but a reflection of a moving front or fronts. Methods for capturing this movement range from different calculations of front velocity [7], to methods that capture the direction of diffusion as related to clusters of human population, network distance or travel distance [8,9].

In this paper, we propose using self-organizing maps (SOMs) to study disease diffusion in space and time. SOMs are a well-known data-mining method, used to cluster and visualize high dimensional data by projecting it into a low-dimensional (typically 2D) space [10]. This projection makes it easier to understand spatiotemporal datasets and the patterns that they might contain [11,12]. In spatial-epidemiology, SOMs are mostly used as a non-linear analytical method to study multivariate patterns [13-15] but here we show that they also enable the integrated analysis of both synchrony and diffusion trajectories. Moreover, this data mining methods is advantageous because it does not require transforming the data to a new “data space” (like wavelets). This greatly facilitates the interpretation of the results as the shape of the epidemiological curve (number of cases as a function of time) is preserved so that the time of infection and intensity (persistence) can be studied for each geographic location.

The detection of synchrony using SOMs is based on the fact that they maintain the topological characteristics of the input data. This ensures that locations with a high level of synchronisation in the timing and intensity are mapped near to each other forming clusters. The study of diffusion trajectories can be achieved by further applying the Sammon’s projection to the previous SOM results. In short, in this study we illustrate the following issues: identification of locations (spatial units) with similar diffusion processes – synchrony (1) and characterization of spatial temporal diffusion patterns – diffusion trajectories (2).

## Methods

### Disease data

To illustrate this study, we used data on eight historical Measles epidemics in Iceland [16]. The epidemics span the period November 1946 (wave 8) to December 1970 (wave 15). Prior outbreaks took place (waves 1–7), but are not included in this research for reason of data incompleteness and re-organization of medical districts. After 1970, outbreaks have different characteristics due to the introduction of mass vaccination.

**Figure 1:**
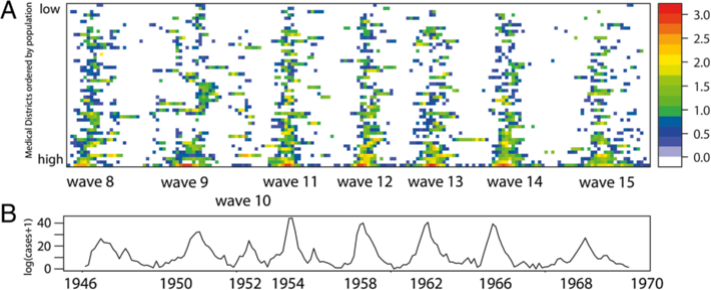
**Measles dataset.** Measles cases in Iceland (1946–1970). **(A)** Spatial distribution of log10 (cases +1) per medical district, with medical districts arranged in ascending order of population size and colour representing epidemic intensity. **(B)** Time series of total number of notified cases for the complete country (log10 (cases +1)).

The data reports monthly Measles cases for each of the 50 medical districts of the country. Figure 1A shows the log transformed Measles cases (log10 (cases +1)) for all of the eight epidemics per medical district, with the medical districts sorted in ascending order of population size. The colour representing the epidemic intensity shows that for all waves, there are only few medical districts with high intensity and that these are always the centres with the higher population (bottom of the graph). The number of cases per epidemic outbreak ranges between 6000 cases for wave 9 and less than 1900 for wave 10 (Figure 1B) [16]. Because of the low number of cases wave 10 is excluded from further analysis. Inter wave periods are evenly distributed showing no significant changes in pattern over the studied time period. All presented analyses are performed on log transformed input data to ensure a normal distribution and are scaled (0–1).

This dataset was selected because Iceland has proven to be an excellent study example for disease diffusion processes for a number of reasons including: the isolation of the country which creates a self-contained system with few external influences; the stability of the population and spatial structure (medical districts), and length of the available time series. This has led to the well-documented and extensively studied Measles dataset [2,17].

### Self-organizing maps (SOMs)

SOMs are a type of un-supervised artificial neural network used to cluster high dimensional data by projecting it onto a low-dimensional lattice. This lattice consists of neurons that are trained iteratively to extract patterns from the input data. These patterns are generalizations of the input data and are referred to as codebook vectors. At the start of the training phase, each neuron is assigned a codebook vector that is updated at each iteration, in such a way that topological properties in the input training data are preserved.

We used the Kohonen R package [18] to train several SOMs following these steps:

a. The size (number of neurons, including number of rows and columns) and type (rectangular or hexagonal) of the SOM lattice were chosen.

b. Each neuron was assigned a random vector of weights or codebook vector (m_*k*_) with the same dimensionality as the input data.

c. Data samples were iteratively presented to the low-dimensional lattice to identify the best matching unit, BMU, which is the neuron that contains the codebook vector that minimizes the Euclidean distance with the data sample at hand. This iterative process is known as training the SOM and each iteration (t) is used to update the codebook vector of the BMU and the neighbouring neurons according to:

(1) $$m_{k}\left(t+1\right)=m_{k}\left(t\right)+\alpha \left(t\right)h_{\mathrm{ck}}\left(t\right)\left(x\left(t\right)-m_{k}\left(t\right)\right)$$

in which m*k* is an n-dimensional codebook vector, α (t) is the learning rate, h_*ck*_*(t)* is the neighbourhood kernel of the BMU neuron and *x* is a randomly chosen input vector from the training dataset.

For the training of the SOM we used a hexagonal SOM lattice, using a standard linearly declining learning rate from 0.05 to 0.01 over 1000 iterative updates. The radius of the neighbourhood kernel uses the starting value of 2/3 of all unit-to-unit distances using a square neighbourhood.

After the training, a secondary clustering can be performed on the SOM lattice, using visual analytics or a different clustering algorithm. Especially when the training lattice is large (larger than the number of clusters needed), a secondary clustering is known to outperform the initial SOM [19]. Secondary clustering can be performed via visual analytics or by using a second clustering algorithm.

A relatively simple way of identifying SOM clusters is by using the U-matrix. The U-matrix displays the Euclidean distance between the codebook vectors of neighbouring SOM neurons. High values in the U-matrix visually separate clusters. However, this method has proven to be difficult, especially with complex datasets. Therefore, several authors have proposed graph-based technics to enhanced the U-matrix for cluster interpretation [20]. Here we used an enhanced U-matrix as proposed by Hamel and Brown [21]. In this method, the centres of the lattice neurons are used as the vertices of a planar graph (a graph without crossing edges). The edges in the graph connect nodes to the neighbouring node with the maximum gradient. In this way, subgraphs are created, that indicate the clustered neurons. When displaying the graph on top of the U-matrix, an easy visual interpretation of the number and composition of the clusters is possible.

Besides the visual identification of clusters based on the U-matrix, a different clustering algorithm can be used for secondary clustering. A range of options exist including k-means and hierarchical clustering [19]. In hierarchical clustering, neurons are first assigned to their own cluster, the distance between clusters is calculated and then, iteratively, the most similar clusters are joined. A disadvantage of these methods is that user has to decide the number of clusters to be found.

After training the SOM and performing the secondary clustering, the third step in the SOM process is the mapping of the data onto the trained SOM, identifying for each input vector the BMU neuron and cluster. The training dataset and mapping dataset can be the same, subsets, or mapping data may consist of new data not included in the training sample. Here, we trained the SOMs with the complete dataset to ensure that all existing patterns are represented in the codebook vectors of the lattice. However, different subsets of the training data are mapped back onto the lattice for evaluation. These subsets correspond to single epidemic waves, making it possible to compare the mapping of the total dataset to the mapping of the individual waves.

The standard way to quantify error for trained SOMs is the *quantization error,* which measures the distance between the mapping data and the codebook vector. In this research, the quantization error is used to evaluate the “goodness of fit” of the mapped data. The smaller the quantization error, the better the mapping.

**Figure 2:**
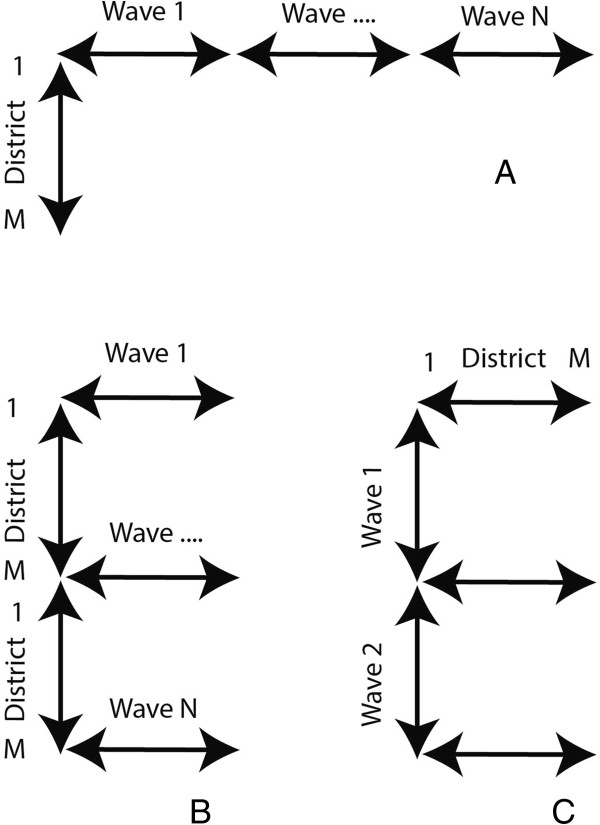
**Data organisation.** Data organisation, Space in Time (SxT) SOM **(A)**, Space over Wave (SxW) SOM **(B)** and Time in Space (TxS) SOM **(C)**.

When applying SOMs for spatiotemporal analyses, the data used for training and mapping needs to be considered in a dual fashion: from a spatial perspective and from a temporal perspective [22,23]. A data organisation of the type space over time (SxT) allows the detection of spatial units (medical districts) that show similar behaviour over time; that are synchronized. Here, we would like to find both synchronies over the total time series and over single waves. Therefore, two different types of space over time datasets are used: the space over time (SxT) dataset where T includes the complete time series (Figure 2A) and the space over wave (SxW) dataset where T covers a single wave (W) (Figure 2B). To study the diffusion of the disease over time, a time over space (TxS) data organization is used (Figure 2C).

### Finding clusters of synchronised codebook vectors

Finding synchronies based on SOMs makes use of the combined ability of the SOM method to produce a generalized prototype vector from the input data and to order these vectors topologically onto a training lattice. Input data vectors that map to the same neuron are synchronised, vectors that map to neighbouring neurons might also be synchronised. This can be identified by performing a second clustering on the SOM lattice grouping neighbouring neurons with similar codebook vectors.

**Figure 3:**
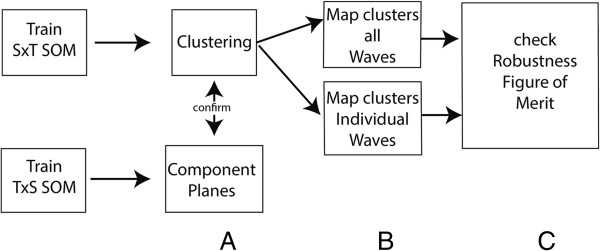
**Flow diagram synchrony.** Flow diagram showing the steps to identify synchrony. **A**- clustering, **B** - mapping of the dataset, **C**- check on the robustness of the clusters.

Detection of synchronies between medical districts is based on an SxT data organisation. The training of the SOM (lattice size 3×4) is followed by a secondary partitioning based on hierarchical clustering (See Figure 3). However, the exact number of clusters is unknown. This is why the clustering is confirmed using the Component Planes of the temporal SOM (Figure 3, step A) with a lattice size of 7×7. Component Planes are slices of the codebook vectors that represent the status of a variable for all the neurons in the SOM lattice. The correlation among variables becomes visible via similar patterns in their component planes. Methods for using Component Planes for correlation hunting have been described previously [24,25].

We can re-organise our dataset in order to construct a dataset were each data vector represents one month (Figure 2 – data organisation (C)). This data organisation is also referred to as Time in Space (TxS). We trained this SOM using a lattice size of 7×7 neurons. When using the TxS SOM, variables represent spatial locations. A component plane in this case, is a representation of all the neurons a medical district has been mapped to, including the frequency. Two spatial locations with identical or similar component planes are correlated. We compared the clustering found with the SxT SOM with the component planes of the TxS SOM to verify the number of clusters. This was done by grouping the component planes of the medical districts per cluster.

After confirmation of the clustering, both the complete dataset and the individual waves are mapped back onto the SxT SOM lattice to determine the BMU (Figure 3 step B). In SOMs, training vector and mapping vector should have an equal length. However, a single wave subset is much shorter than the complete time-series. Thus, subsets of input vectors were created by combining a Nodata matrix with subsets of the scaled input data. This is possible because SOMs allow for “missing data”.

When training the SOM with the complete dataset and mapping back single waves, clustering found in the complete dataset may differ from the clusters of individual waves. The robustness of the clusters was checked via the so-called figure of merit [26]. The figure of merit (M(v)) measures the extent to which the clustering for the subsamples (individual waves) corresponds to the clustering of the complete dataset for the variable or variables v, in our case the disease incidence. Mapping can be presented as an *N × N* mapping matrix Ƭ*ij* in which Ƭ*ij* = 1 when two medical districts are mapped to the same neuron or cluster, and Ƭ*ij* = 0 when mapped to different neurons or clusters. The figure of merit is based on the comparison of mapping matrices of the resamples Ƭ(μ) and the original matrix Ƭ per subset (w), in our case a wave:

(2) $$M\left(v\right)=\frac{\Sigma_{w}\left(\frac{\delta \tau_{\mathrm{ij}},\tau_{i,j}^{\mu }}{\tau_{i,j}}\right)}{w}$$

M(v) is used to compare the mapping of all the subsamples with the mapping of the complete dataset by counting the number of times the same mapping occurs in both samples and dividing this by the total number of subsets. M(v) = 1 indicates a perfect score.

### SOMs for identifying diffusion patterns

**Figure 4:**
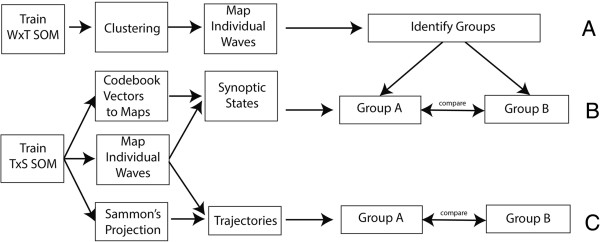
**Flow diagram spatial diffusion.** Flow diagram showing the steps for the identification of diffusion. **A** - grouping of similar waves. **B** - comparison of synoptic states of the groups identified under A. **C**- mapping of diffusion trajectories of all waves onto the Sammon's Projection, and comparison of the trajectories of the groups identified under A.

SOMs can also be used to identify diffusion trajectories. For this, we followed two steps: First, we grouped epidemics based on their diffusion pattern. Next, we visualised the synoptic states and created diffusion trajectories (Figure 4).

### Grouping waves with similar diffusion patterns

Grouping of epidemic waves with similar characteristics is done based on the SxW SOM (Figure 2 – data organisation (B)). For the SxW SOM, codebook vectors represent a medical district during a single epidemic. This SOM maintains the epidemic curve in the purest form, and allows for a high level of topological consistency. After training (lattice size 7×7 neurons), a secondary grouping is performed using the enhanced U-matrix method, and the individual waves are mapped back onto the SOM lattice (Figure 4(A)).

A limitation of the SOM algorithm is that all input vectors should be equal in length. As this data organisation is per wave, and waves cover different time periods, the vectors are aligned at the beginning of the wave, and zero values are added to shorter outbreaks, to ensure equal vector length.

Grouping of waves is found by comparing the mapping of the individual waves on the SOM lattice.

### Sequence of synoptic states

A synoptic state is a pattern that spatially characterises a diffusion state. Each wave can be represented as an ordered sequence of synoptic states. The number of states per wave is variable. This sequence provides information on the speed and on the direction of spread. The workflow for generating trajectories of synoptic states is shown in Figure 4B.

In order to retrieve synoptic states, a SOM is trained using the TxS SOM (Figure 2 – data organisation (C)). In this case each data vector represent one month (variables represent the medical districts). After training the SOM, codebook vectors are translated into a GIS map (Figure 4 – (B)). This can be done because the variables of each codebook vector represent a sequence of medical districts. By transposing the codebook vectors (to SxT) and visualising them in a GIS, each neuron (codebook vector) of the SOM lattice can be shown as a GIS map.

The data is now mapped back onto the SOM. For each month a mapping to a codebook vector is determined. After grouping successive months with the same mapping (within the same wave), a sequence of maps is retrieved, this composes the ordered sequence of synoptic states per wave.

States differ in duration. The speed is represented as the number of states and the duration of each synoptic state (the number months mapped to the state). The direction of spread can be derived from the maps via visual comparison of the states.

### Sammon’s trajectories

Alternatively, the direction of spread can be evaluated by mapping trajectories on the Sammon’s projection (Figure 4 – (C)). The method of combining the analysis of synoptic states and Sammon’s trajectories has been previously used by Zurita-Milla et al. [27]. Yet, here we applied it in combination with mapping back subsets on point data.

This trajectory is constructed on a “TxS*”* SOM, in which each vector in the input dataset represents an epidemic month. This is the same SOM and the same mapping as used for the synoptic states.

To describe the diffusion path, the Sammon’s projection is used to visualise the SOM codebook vectors in 2-D space. The Sammon’s projection aims to minimize the following error function:

(3) $$E=\frac{1}{\sum_{i<j}d_{\mathrm{ij}}^{*}}\sum_{i<j}\frac{{\left(d_{\mathrm{ij}}^{*}-d_{\mathrm{ij}}\right)}^{2}}{d_{\mathrm{ij}}^{*}}\;$$

in which d*_ij_ is the Euclidean distance between the vectors *i* and *j* in the input space (the codebook vectors), and d_*ij*_ is the corresponding distance in the output space (the Sammon’s coordinates).

Like this, each codebook vector in the SOM lattice can be projected to a 2D space. The diffusion trajectory is the vector that depicts the “sequence of movement” over the SOM lattice. Arrows connect neurons in the order in which they are mapped and the shapes of different epidemic vectors can be compared to reveal (dis)similarity between diffusion patterns of different waves.

The R script used to perform the analysis discussed here is available as Additional file 1.

## Results

### Spatial synchrony

**Figure 5:**
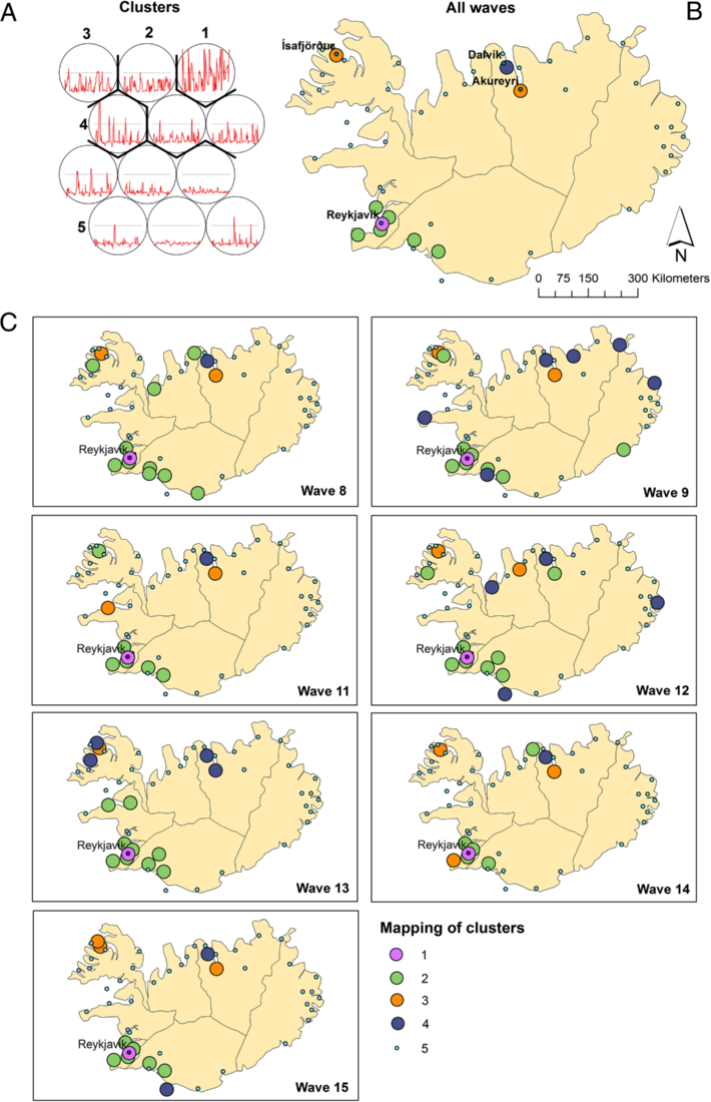
**Results Synchrony. (A)** SOM lattice showing hierarchical clusters (black lines). Numbers indicate the order of the neurons. **(B)** GIS mapping of the complete dataset (all waves), and the individual waves. **(C)** Numbers in the legend correspond to the numbers of the neurons in the SOM lattice.

Identification of spatial synchrony was performed as described in Methods - section “spatial synchrony”. After training, the SOM lattice was partitioned into five clusters (Figure 5A - Lattice with clusters) identifying neuron 12 as cluster 1, neurons 8, 9 and 11 as cluster 2, neuron 10 as cluster 3, neuron 7 as cluster 4 and neurons 1–6 as cluster 5.

**Figure 6:**
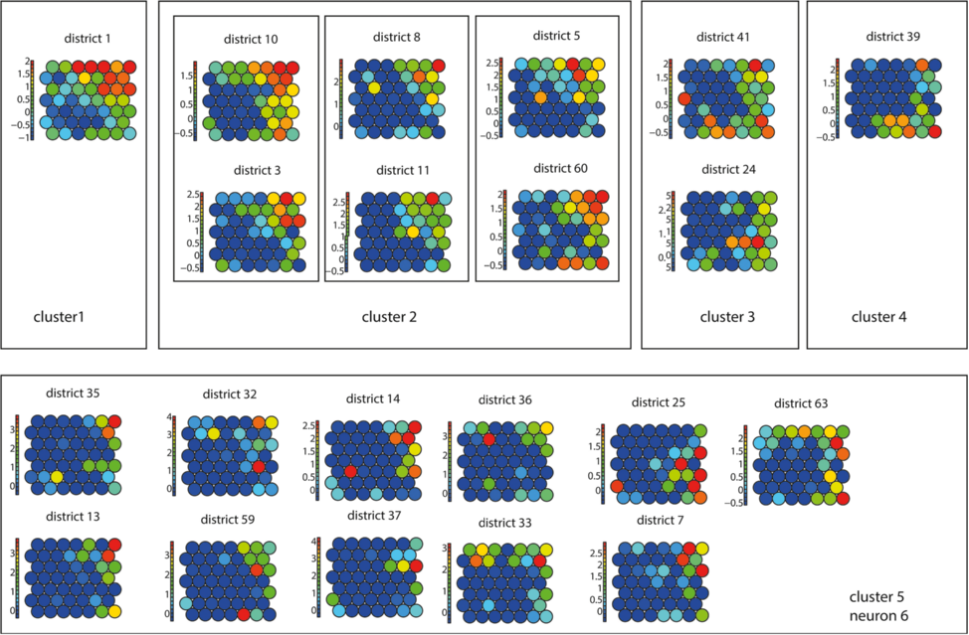
**Component planes.** Component planes of a (TxS) SOM, organized by hierarchical cluster.

The identified clusters were verified using the component planes of the TxS SOM (Figure 6). When comparing the component planes of the clusters, it can be confirmed that districts mapped to the same cluster show good correlation. In our experiment, including one extra class in the hierarchical clustering would lead to neuron 6 being identified as a separate class. When visualising the component planes of this class (Figure 6 – cluster 5), it turns out that the patterns in this group are not very prominent and the group is not very homogeneous compared to the other classes.

The mapping of both the complete dataset and of the individual waves was visualised in a GIS (Figure 5B and 5C). The results of the mapping for the complete time series, revealed that cluster 1 represents the medical district in which Reykjavik (the most dominant city in the process) is located, and a group of medical districts mapped to cluster 2, surrounding Reykjavik, are highly synchronised. On the SOM lattice this cluster is adjacent to cluster 1. In the north of Iceland three more medical districts were identified (mapped to clusters 3 and 4) that are potential regional diffusion centres. They have a relatively high incidence rate but are topologically further away from Reykjavik on the SOM lattice. On further examining it is revealed, that these correspond to the areas of Ísafjörδar and Akureyri. By examining the codebook vectors of the SOM lattice, neurons 1–6 are grouped into one large cluster (cluster 5). The codebook vectors of these neurons show that these represent medical districts with lower frequencies.

When comparing the total mapping with the mapping of the individual waves (Figure 5) we see that in each of these waves more local medical districts are mapped to clusters 1–4 (indicating a role in the diffusion process). This shows that there is a group of medical districts that are important in all outbreaks, but also medical districts that play a role in the diffusion process of single epidemics. For incidental mapping to cluster 2 (highly synchronised with Reykjavik) we see several additional mappings in the northern parts in almost all waves. Incidental mapping to clusters 3 and 4 may indicate (second level) diffusion synchrony with local northern and north western centres or a different diffusion pattern. Especially wave 9 has many medical districts mapped to cluster 4 throughout the island. This can be an indication of a different direction of diffusion.

The quantization error is the average distance between the input vector and the BMU. The results are shown in Table 1. Quantization error for the complete dataset is high (77.11). After mapping the individual waves, this value improves to 42.33-73.56. The error for the complete dataset is high because it is difficult to match a vector over the total length of the time series. Values improve however (become smaller) when matching only parts of the time frame.

**Table 1:** Quantization error

**Wave(s)**	**All**	**8**	**9**	**11**	**12**	**13**	**14**	**15**
**SxT SOM**	77.11	46.89	76.72	60.26	50.90	58.80	51.20	54.38
**TxS SOM**	11.40	11.76	11.81	10.29	12.67	12.86	9.04	10.88
**WxT**	6.08	4.20	10.40	3.88	3.96	6.50	4.87	8.91

Quantization errors for all SOMs.

To test the robustness of mapping back of individual waves the figure of merit M(v) was calculated (Table 2). This figure expresses the number of medical districts that were mapped to the same cluster for the mapping of the complete dataset and also for the mapping of the individual waves. The average over the combined waves was 0.82 (scale 0–1), indicating robust clusters over the temporal period.

**Table 2:** Figure of merit

**Wave**	**8**	**9**	**11**	**12**	**13**	**14**	**15**
**m(v)**	0.77	0.7	0.94	0.72	0.77	0.94	0.9

Results figure of merit for mapping of individual waves.

### Spatiotemporal diffusion and trajectories

#### ***Grouping waves***

**Figure 7:**
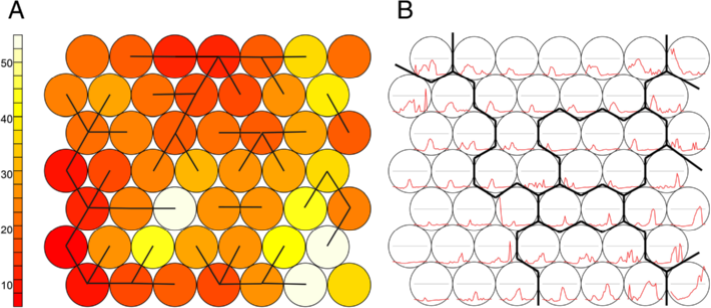
**Clusters SxW SOM.** Enhanced U-matrix, with light background colours indicating high values, dark colours indication low values and graph lines indicating the clusters **(A)**. Lattice with codebook vectors and cluster lines **(B)**.

The grouping of epidemic waves was performed as described in Methods - section “Grouping waves”. This experiment produced a SOM with a high level of topological consistency between the neurons (Figure 7A). Visually, interpretations like “early” (top right), “middle” (top left), “late” (bottom right), and “high” (edges) or “low” (middle) intensity can be given to the neurons. By enhancing the U-matrix, ten clusters were identified (Figure 7B).

**Figure 8:**
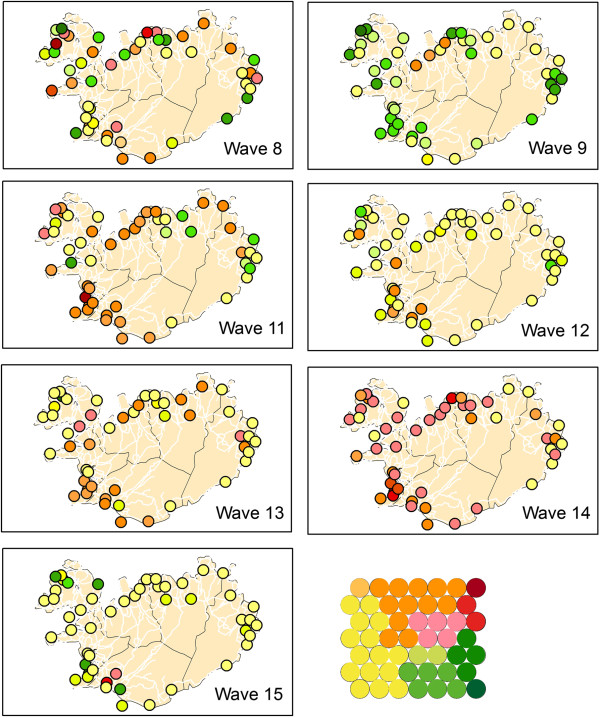
**GIS mapping SxW SOM.** GIS mapping using colour coding for the clusters.

The partitioning of the SOM lattice into clusters can be translated into a heat key and the data can be visualised in a GIS. Where red colours represent “early”, yellow colours “middle” and green colours represent “late”. Figure 8 shows the mapping of the individual waves using this heat key. Comparison of the GIS maps revealed that waves 11 and 14 are fast (early) developing waves (primarily red coloured), waves 12 and 15 are primarily yellow, meaning their spread is of medium speed, and wave 9 is a late developing wave (green coloured). However, interpretation of these maps is “intuitive” (it depends on the human interpretation of the colour scheme). Hence, it is easier to evaluate the results by mapping directly onto the SOM lattice.

**Figure 9:**
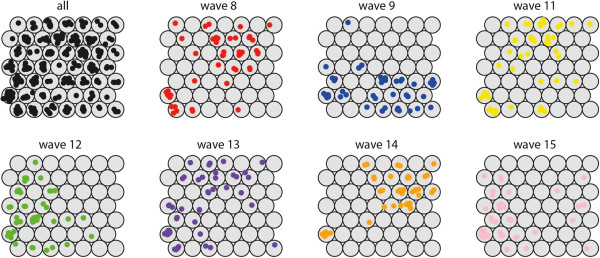
**Mapping SxW SOM on SOM lattice.** Mapping of the Medical Districts on the SOM lattice. Each dot representing one medical district.

This leads to the results shown in Figure 9. After mapping the complete dataset, each wave was mapped back individually. Most waves do not have mapped samples for all neurons or clusters, but the mapping is grouped to a particular area of the lattice. Similar waves should be projected to the same clusters of the lattice.

Two groups of epidemics were identified. These are the fast developing (early) epidemic waves 8, 11, 13 and 14 (Group A) that have many medical districts mapped to the upper right hand of the lattice, and the slow developing (late) epidemics, waves 9, 12 and 15 (Group B) that show a mapping to the lower and left part of the SOM lattices. The quantization error for this experiment, when mapping back the complete dataset, is 6.08 (Table 1). This shows that for this experiment, the distance between the codebook vectors and the data vectors was much smaller compared to the mapping of the synchrony experiment. This was to be expected as single waves were used.

#### ***Synoptic states***

**Figure 10:**
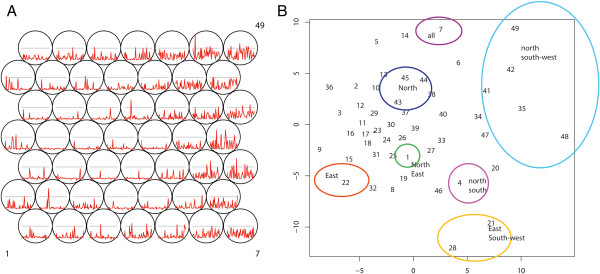
**Codebook vectors and Sammon’s Projection TxS SOM.** Lattice showing codebook vectors, with neuron 1 in the lower left corner, numbering in rows, from left to right and bottom to top, ending with neuron 49 in the top right hand corner **(A)** and Sammon’s Projection with interpretation **(B)**.

**Figure 11:**
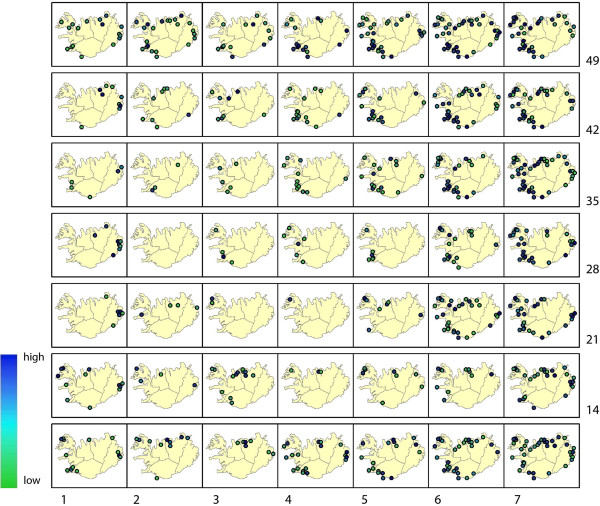
**Lattice converted to GIS maps.** Lattice showing the codebook vectors as maps of synoptic states. Numbering from left bottom corner (1) to the top right (49), in rows from left to right.

This experiment was conducted on a Time in Space (TxS) SOM as explained in Methods - section “Synoptic states”. The trained lattice is shown in Figure 10A. For this type of SOM, each codebook vector represents one specific spatial pattern, so the SOM lattice can be translated into a lattice of GIS maps (see Figure 11).

When we conducted an interpretation of these maps, it turns out that neuron 23 represents a static state of almost no disease occurrence. Around this neuron, we identified synoptic states that represent disease occurrence in certain (combinations of) compass points, with the top right of the lattice corresponding to infection in the north and south-western parts of Iceland, and the lower left of the SOM lattice corresponding to infection in the eastern and northern parts of the island.

**Figure 12:**
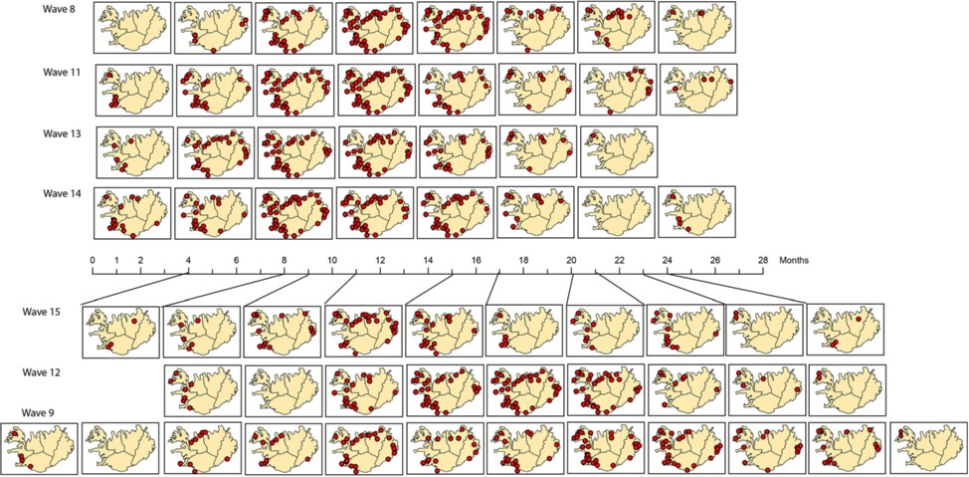
**Trajectories of synoptic states.** Representation of the waves as a sequence of synoptic states.

Next, the codebook vectors of each wave were mapped as an ordered synoptic spatial time series in a GIS (Figure 12). This way, a sequence of maps per epidemic wave can be constructed to reflect the spatialtemporal patterns found in each epidemic wave. When comparing the patterns of Group A – consisting of waves 8, 11, 13 and 14 – we noticed that these patterns are all about equal in length, and are spatially very similar. This group seems to consist of fast developing waves. Group B – waves 9, 12 and 15 – shows more diversity in number of synoptic states and in diffusion pattern. Information about the number and duration of synoptic states can also be found in Table 3. Number of states for Group A ranges from 9–10, for Group B from 11–14. Duration of each state ranges from 1–6 months. Group B waves have a longer duration of the first two states.

**Table 3:** Synoptic states

			**Number of months per state**													
**Wave**	**Group**	**# states**	**1**	**2**	**3**	**4**	**5**	**6**	**7**	**8**	**9**	**10**	**11**	**12**	**13**	**14**
**8**	A	9	1	2	1	2	1	3	3	3	6					
**9**	B	14	4	1	1	3	2	1	2	3	1	2	3	1	2	1
**11**	A	10	1	3	2	2	2	2	2	2	4	3				
**12**	B	11	4	2	2	1	1	2	3	1	2	2	1			
**13**	A	9	2	2	3	1	4	2	3	8	1					
**14**	A	9	2	2	3	1	4	2	3	8	1					
**15**	B	12	3	4	1	2	4	2	3	1	2	1	1	3		

Number and duration (in months) of synoptic states per wave.

#### ***Sammon’s trajectories***

**Figure 13:**
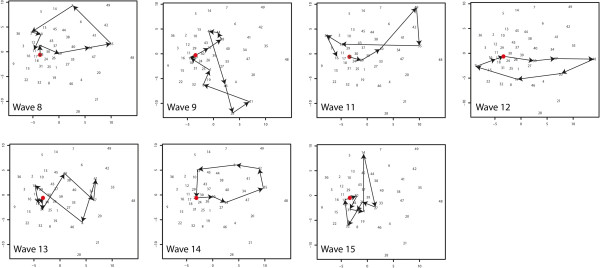
**Trajectories on Sammon’s Projection.** Trajectories of spatiotemporal diffusion on the Sammon’s Projection.

For a further analysis of the diffusion direction, the diffusion trajectories were projected as a Sammon’s projection (Figure 13). This experiment is described in section “Sammon’s Trajectories” of the Methods section. The data organisation and the SOM lattice (see Figure 10A) were the same as for the previous experiment. The Sammon’s projection of the lattice is shown in the same figure (Figure 10B). In this projection the distance between the vectors is explicitly mapped, but the topological relationships are not necessarily maintained. Numbers in the Sammon’s projection refer to the numbers of the neurons. As can be observed, the Euclidean distance between the neurons in the top right hand of the SOM lattice (numbers 35, 42, 48, 49) is relatively large.

For each wave, the “trajectory of diffusion” was mapped onto the Sammon’s projection (Figure 13) and the trajectories were compared. Waves 8 and 14 both have a trajectory starting in neuron 23, moving in a circular fashion to the right hand side of the figure (reaching neuron 35 as one of their peak stages) to return to neuron 23. Their diffusion pattern is strikingly alike. This diffusion corresponds to the sequence of “infection in the Reykjavik area” followed by “infection in the Reykjavik area and the north”, and returning via infection for example infection in the east back to neuron 23.

Waves 11, 12 and 13 have trajectories with similar characteristics compared to the previous group. Their trajectories also follow a circular path from neuron 23 to the upper-right hand side of the graph indicating similar diffusion. However the lines of waves 11 and 13 show cross-overs and wave 12 shows an opposite direction. Cross-overs occur when a fast spread (or decline) to all areas takes place. Where waves 8 and 11 decline to the north, the opposite direction of wave 12 is triggered by a decline to the north and south of the island. However, these diffusion trajectories do not differ significantly from the trajectories of wave 8 and 14.

Wave 9 and 15 have the strongest deviation from the general pattern. For the interpretation of these results we mapped the compass directions onto the Sammon’s projection (Figure 10B). Wave 9 and 15 show strong vertical trajectories. These correspond to a different spatiotemporal diffusion pattern. For Wave 9 this is a pattern from neuron 45 to 28 (spread starting in the north) and for Wave 15 from (1, 14, 27) from the east, via the north to the western parts of the island.

## Discussion

The use of SOMs, combined with Sammon’s projection, has enabled us to identify synchrony between locations (medical districts), and to map diffusion trajectories of a time series of epidemic waves revealing their spatiotemporal diffusion patterns. This integrated approach was carried out using three different data organisations (in space and time). Training the SOM on the complete time series and mapping back individual waves has shown to be a simple but effective way to compare general spatiotemporal diffusion patterns for a complete time series with patterns of individual waves. Results found are consistent with results found for the same epidemics, using different methods.

The synchrony experiment revealed a number of medical districts that form the diffusion structure for all of the waves and, additional medical districts that only play a role in some waves. The medical districts that were identified as forming the stable structure all have a large number of inhabitants and the centres in the north and north west are connected to Reykjavik via domestic air travel. The identified medical districts show a great similarity to the structure found by Cliff et al. [17], in their quarterly lag maps of geographical spread, first and second quarter. They are also consistent with epidemiological theory (hierarchical diffusion models).

The research identified two different groups of epidemics with fast developing (group A) and slower developing waves (Group B). These groups were identified using the SxW SOM but the synoptic state experiment, based on the TxS SOM revealed the same grouping. Similar results were found by Cliff et al. [2] who found for Group A mean lag time in months of respectively 8.42, 9.05, 10.54 and 5.85 months and for Group B a mean lag time of 15.45, 11.29 and 13.32 months.

Experiments also showed that the fast developing waves (Group A) show considerable similarity in their spatiotemporal patterns. They all spread from Reykjavik to the north-eastern areas. Group B waves cannot be characterized by a single direction of spread; yet, the diffusion trajectories show that the spread of wave 9 and 15 does not follow the Reykjavik pattern. Findings were compared to Cliff et al. [2] that describe the spread of wave 9 as being confined to the northern parts of the island. For wave 15 the same source notes that it was slow moving, that it started in the south, but the difference in diffusion pattern, was not reported.

Two methods were used for the clustering of the trained SOMs: the enhanced U-matrix and hierarchical clustering combined with a validation based on component planes of a temporal SOM. Although both methods lead to reliable results, the enhanced U-matrix is advantageous because it does not require the user to determine the number of clusters. However, other enhancement methods for the U-matrix exist, for example methods including the second best matching neuron [20]. These may be worth further exploring.

The proposed method was used on a time series of seven epidemic waves and a relatively small number of spatial locations. Spatially this method is scalable without any problems. However, there are probably a minimum number of waves needed to come to a reliable mapping of the individual outbreaks. If the diversity in the training dataset is too small this may lead to problems. The synchrony experiment was tested with shorter time series including fewer epidemics. A series of 4 outbreaks still gave a reliable result for our case study, but this may depend on the complexity of the dataset.

Iceland is a small island with only one larger city and it is clear that this city is the “motor” of the diffusion mechanism. In this regard, the selected dataset is ideal for testing new methods (also because there are seven epidemic waves available). However, it would be interesting to test the proposed method in a much more heterogeneous environment (with more large cities and a more complex diffusion pattern). For this study all medical districts were included, but some of these centres represent areas with low population. When working with larger datasets, removal of sparsely populated areas may be an option.

SOMs are relatively easy to train and combine with other visualisation methods to enhance their analytical possibilities. However, results are very sensitive to values of input parameters (size and shape of the training lattice, number of iterations, type of initialization), and deriving meaningful information can be challenging [28]. The method as applied here, therefore focusses more on comparison then on absolute characterisation of patterns.

Besides Measles, this method is potentially useful to explore and understand spatialtemporal diffusion patterns of other infectious diseases (e.g. Influenza, Pertussis) as SOMs can deal with large datasets as well as with missing data. This understanding might lead to new paradigms of modelling and validating spatially explicit disease models based on reproducing observed diffusion patterns.

This method can also be used for real time disease mapping. As data from partial outbreaks can be mapped back, comparison of diffusion patterns with previous waves may lead to early indications of the characteristics of an epidemic and, thus, help to design intervention actions. When linked to systems for Volunteered Geographic Information, web-based monitoring networks a fully automated analysis may be an interesting option.

## Conclusions

In this paper we proposed a SOM-based method to analyse spatiotemporal diffusion of infectious diseases. The method is based on training a SOM for a larger time-series (including multiple waves) and mapping back individual outbreaks for characterisation and comparison. Via a number of experiments we showed how this method can be applied for finding synchronies between spatial locations and for comparing spatialtemporal diffusion patterns of different epidemics.

We also demonstrated how different types of data organisation (in space and time) can help to reveal different information. Several types of secondary clustering (hierarchical, enhanced U-matrix and Component planes) were shown, that can be used to improve the SOMs performance. The integration of SOMs with other visualisation techniques, especially Sammon’s Projection and GIS was used to detect, interpret and visualise spatial temporal patterns.

Results of the method are consistent with diffusion patterns found using other methods; this makes SOMs an interesting alternative, worth further exploring. For instance, by applying it to a larger dataset in a more dynamic geographic environment, by coupling it to a spatially-explicit disease model or by using it for near-real time disease monitoring.

## Competing interests

The authors declare that they have no competing interests.

## Authors’ contributions

EWA and RZM designed the experiment together, EWA performed the data analysis, EWA and RZM analysed the results and wrote the paper together. Both authors read and approved the final manuscript.

## Supplementary Material

Additional file 1 **The R script used to perform the analysis discussed in this paper.** This file shows in detailed steps how the analyses were performed, including comments with explanatory details. Click here for file
